# Regulatory T cell-mediated anti-inflammatory effects promote successful tissue repair in both indirect and direct manners

**DOI:** 10.3389/fphar.2015.00184

**Published:** 2015-09-02

**Authors:** Hong Lei, Katharina Schmidt-Bleek, Anke Dienelt, Petra Reinke, Hans-Dieter Volk

**Affiliations:** ^1^Berlin-Brandenburg Center for Regenerative Therapies, Charité University Medicine Berlin, Berlin, Germany; ^2^Institute for Medical Immunology, Charité University Medicine Berlin, Berlin, Germany; ^3^Julius Wolff Institute, Charité University Medicine Berlin, Berlin, Germany; ^4^Department of Nephrology and Intensive Care, Charité University Medicine Berlin, Berlin, Germany

**Keywords:** inflammation, tissue regeneration, regulatory T cells, osteoblasts, purinergic signaling

## Abstract

Regulatory T cells (Tregs) offer new immunotherapeutic options to control undesired immune reactions, such as those in transplant rejection and autoimmunity. In addition, tissue repair and regeneration depend on a multitude of tightly regulated immune and non-immune cells and signaling molecules. There is mounting evidence that adequate innate responses, and even more importantly balanced adaptive immune responses, are key players in the tissue repair and regeneration processes, even in absence of any immune-related disease or infection. Thus, the anti-inflammatory and anti-apoptotic capacities of Treg can affect not only the effector immune response, creating the appropriate immune environment for successful tissue repair and regeneration, but growing evidence shows that they also have direct effects on tissue cell functions. Here we summarize the present views on how Treg might support tissue regeneration by direct control of undesired immune reactivity and also by direct interaction with non-immune tissue cells. We describe tissue-resident Treg and their specific phenotypes in skin, visceral adipose tissue, and skeletal muscle. In addition, we touch on the topic of osteoimmunology, discussing the direct interactions of Treg with bone-forming cells, such as osteoblasts and their mesenchymal stromal cell (MSC) progenitors—a field which is under-investigated. We hypothesize a cross-talk between Treg and bone-forming cells through the CD39–CD73-(adenosine)-adenosine receptor pathway, which might also potentiate the differentiation of MSCs, thus facilitating bone regeneration. This hypothesis may provide a road map for further investigations on the cross-talk between the immune and the skeletal system, and also enable the development of better strategies to promote bone repair and regeneration.

## Diversity of Tregs

Regulatory T cells (Tregs) are a specialized subpopulation of T cells that can control undesired immune responses. They play a central role in maintaining homeostasis within the immune system, including both innate and adaptive immune networks, and also regulate inflammatory processes as those seen with tissue injury, transplant rejection, and autoimmunity.

As Tregs can function in secondary lymphoid organs, as well as the periphery, specialized subsets with distinct molecular mechanisms have developed with respect to these differing microenvironments. The thymus-derived Treg (tTreg) or so-called natural Treg is the dominant form of Treg, and shows a high expression of the transcription factor forkhead box P3 (FoxP3), which is the master control gene of Treg function and Treg development in the thymus (Hori et al., 2003; Roncador et al., 2005). FoxP3^+^ Treg can furthermore be induced from naïve conventional T cells (Tconv) in the presence of specific cytokines and low amounts of antigens in the periphery (Furtado et al., 2002; Apostolou and von Boehmer, 2004; Curotto de Lafaille et al., 2004), to yield the so-called induced Treg (iTreg). However, only tTreg express a demethylated region within the FoxP3 promoter, which stabilizes their phenotype. Type 1 regulatory T cells (Tr1 cells) are another type of adaptive Treg produced in the periphery, which express FoxP3 weakly, or not at all, but secrete IL10 and express granzyme B to kill myeloid antigen-presenting cells (Groux et al., 1997; Magnani et al., 2011). All these Treg can control the immune responses of different cell types with several specific, partially overlapping mechanisms.

**FIGURE 1:**
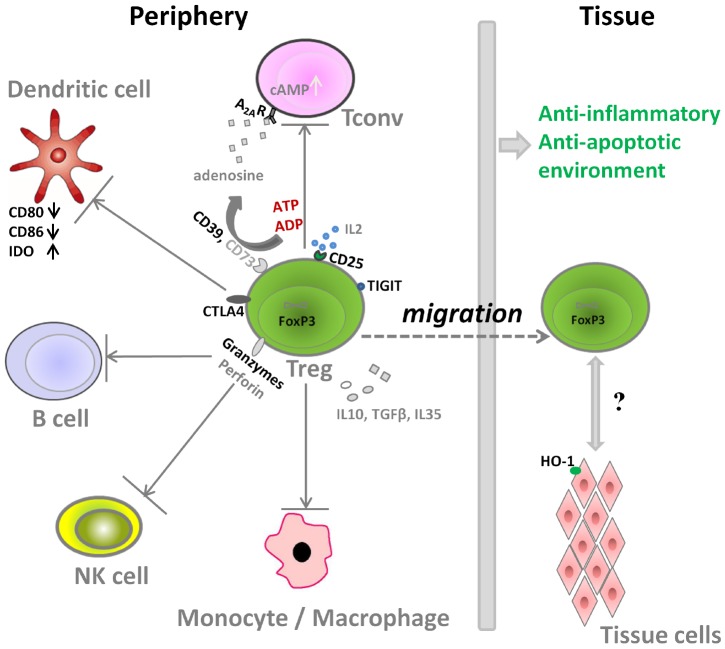
**Molecular mechanisms used by Treg for the suppression of immune cells**.

Nevertheless, tissue-resident Treg express different phenotypes, chemokine receptors, and T-cell receptors (TCRs), depending on their tissue location. The characteristics of tissue-resident Tregs have been thoroughly reviewed elsewhere (Burzyn et al., 2013a). Here, we summarize the phenotype and function of the best studied tissue resident Treg. Characteristics of Treg in skin, visceral adipose tissue (VAT), skeletal muscle tissue, and solid tumor in non-lymphoid tissues are briefly described in Table 1. These Treg promote the tissue repair process through the control of immune responses of T cells and other immune cells infiltrating the tissue (Figure 1), but also through the regulation of some non-immune pathways. For example, VAT Treg express peroxisome proliferator-activated receptor gamma (PPAR-*γ*), which is mainly present in adipocytes as a “master regulator” of their differentiation and glucose metabolism. VAT Treg can also activate the scavenger receptor CD36 expression to take-up lipids and promote adipogenesis, and PPAR-*γ* also interacts with FoxP3 to up-regulate Treg signature *in vitro* (Cipolletta et al., 2012). In skeletal muscle, Burzyn et al. (2013b) found that a distinct Treg population can potentiate the muscle repair process through expression of the epidermal growth factor amphiregulin, which acts directly on muscle satellite cells *in vitro* and improves muscle repair *in vivo* in mice.

**TABLE 1:** **Tissue resident Treg express different phenotype and function in different tissues**.

**Resident tissue**	**% of local resident CD4 T cells**	**Treg phenotype and characteristics**	**Treg function**	**Reference**
Skin	50–60%	→ Express effector-memory phenotype → Express CCR10	→ Control inflammation and keep immune homeostasis in skin	Xia et al. (2014)
Visceral adipose tissue (VAT)	>50%	→ Express CCR1, CCR2, CCR9 → Secrete IL10 → Distinct TCR with lymphoid-organ Treg → Express PPAR-*γ* Express CD36	→ Control CD4 and CD8 Tconv in adipose tissue → Control co-resident pro-inflammatory macrophages and monocytes → Regulate adipocyte differentiation and promote Treg survival and frequency → Take up lipids	Feuerer et al. (2009), Cipolletta et al. (2012)
Skeletal muscle	50–60%	→ Up-regulate IL10 production → Express amphiregulin → Skewed TCR different with muscle Tconv TCR	→ Control the switch of pro-inflammatory to anti-inflammatory response in injured muscle → Act directly on muscle satellite cells and improve muscle repair	Burzyn et al. (2013a,b)
Solid tumor in non-lymphoid tissue	30–50%	→ Highly express CCR10, CTLA-4 → Secrete immune suppressive cytokines IL10 and TGF-β	→ On immune targets: facilitate tumor growth → On non-immune targets: pro-angiogenic effect	Facciabene et al. (2011), Tan et al. (2011)

## Homing and Accumulation of Tregs at Sites of Injury

Tissue regeneration is not only influenced by Tregs residing within the injured tissue, but also by recruitment of Tregs to sites of injury, which can lead to a 30–60% increase in cell number compared to local resident CD4 T cells. This is counterintuitive, given that most circulating Treg in murine models express the phenotype of naïve or central-memory T cells, which can only home to lymphoid tissues via CD62L/CCR7. Nevertheless, mounting evidence has been published that Treg can promote the regeneration process directly in the tissue (Burzyn et al., 2013b), potentially by homing facilitated by their heterogeneous expression of the naïve/memory marker CD45RA, and some homing receptors.

**FIGURE 2:**
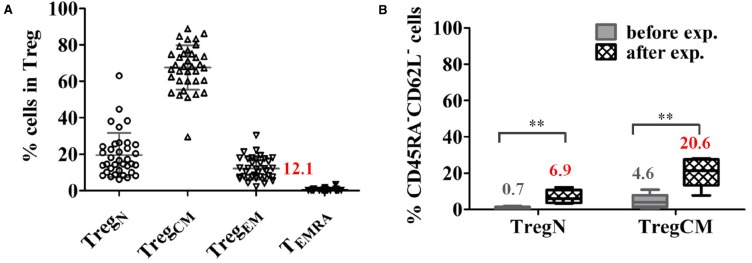
**A proportion of Treg express an effector-memory phenotype in healthy donors. (A)** Proportions of Treg subsets in total Treg in healthy donors (mean ± SD, *n* = 36, age 19–87 years). (B) A significant proportion of expanded naïve (TregN) and central-memory Treg (TregCM) converted into EM (CD45RA^–^CD62L^–^) phenotype upon 3 weeks of expansion with poly-clonal stimulation in the presence of rapamycin and interleukin 2. The mean frequency of cells expressing an EM phenotype is indicated for each cell type before and after expansion. (*n* = 5), paired *t*-test, taken from Lei et al. (2015) **p < 0.01.

The recruitment of Treg from the periphery to various non-lymphoid tissue is mainly associated with the following consecutive/overlapping issues:

(i) Expression of chemokine receptors on Treg, like CCR6, CXCR3, CCR4, CCR7, and CCR10 that support attraction of Treg to the specific tissues and execute rapid intra-tissue regulation (Grindebacke et al., 2009; Duhen et al., 2012; Chow et al., 2015). We recently demonstrated that about 10% Treg in the peripheral blood of healthy donors and patients awaiting for kidney transplantation express an effector-memory phenotype (CD45RA^–^CD62L^–^) allowing migration into inflamed tissues without further activation (Figure 2A). Upon *in vitro* expansion, a significant proportion of both naïve (CD45RA^+^CD62L^+^) and central-memory (CD45RA^–^CD62L^+^) Treg shift their phenotype into effector-memory like (Figure 2B), which could facilitate further migration of Treg into tissues when the expanded Treg products are transferred into patients (Lei et al., 2015).

(ii) Exposure to tissue antigens can induce clonal expansion of Treg, which could result in the increase of intra-tissue Treg and bias of the TCR repertoire of Treg. Interestingly, we also observed that effector-memory Treg express a biased and less polyclonal TCR repertoire in the peripheral blood as shown by next-generation sequencing (Table 2; Lei et al., 2015). These data support the view that an (auto)antigen-driven expansion of memory-effector Treg can contribute to the control of intra-tissue inflammation/regeneration.

**TABLE 2:** **Coverage of the TCR repertoire by the top 20 clones in each Treg subset repertoire as shown by next-generation sequencing**.

**Donor**	**TregN (%)**	**TregCM (%)**	**TregEM (%)**
D1	5.6	8.8	35.2
D2	2.9	15.7	38.5
D3	9.5	13.9	40.9
D4	2.7	8.1	30.3
D5	7.8	19.7	36.1
Median (interquartile range)	5.6 (2.8–8.7)	13.9 (8.5–17.7)	36.1 (32.8–39.7)

(iii) Exposure to tissue antigens in a tolerogenic environment can also induce naïve FoxP3^–^ Tconv to express FoxP3 and to become iTreg.
(iv) The acquisition of the tissue-specific phenotype of tissue resident Treg can facilitate their adaption and survival ability in the tissue (Rosenblum et al., 2011; Burzyn et al., 2013a; Lehtimaki and Lahesmaa, 2013; Gratz et al., 2014).

## Interaction Between Tregs and Immune Cells

Aside from the direct stimulatory effects of Tregs on tissue specific cells with regenerative properties (e.g., muscle satellite cells), Tregs can also impact tissue regeneration by modulation of local inflammation after injury. The molecular mechanisms that Treg use for the suppression of immune cells are shown in Figure 1. On one hand, Treg can secrete several immunosuppressive cytokines including transforming growth factor beta (TGFβ), IL10, and IL35 to control the immune responses. Treg are also reported to induce M2 macrophages, another key player in the tissue repair and regeneration, partly thorough IL10 and TGFβ pathway (Liu et al., 2011a; Weirather et al., 2014; Tan et al., 2015). On the other hand, Treg also express several important molecules to interact with other cells types and to counteract their activity (Figure 1):

(i) Tregs express high amounts of CD25, the *α*-chain of IL2 receptor, leading to IL2 consumption and thus inhibition of Tconv activation and proliferation (Baecher-Allan et al., 2001; de la Rosa et al., 2004).
(ii) Tregs express CD39, an ectonucleotidase, that can facilitate the crosstalk between Treg and CD73-expressing cells to hydrolyze ATP (Schuler et al., 2014; Zhang et al., 2015), which is released into areas of tissue injury upon apoptosis of cells. The cleavage of ATP by Treg through the ectonucleotidase CD39 (ATP → ADP/AMP) and CD73 (AMP → Adenosine) would result finally in adenosine formation. Through adenosine receptor activation (primarily adenosine receptor A2A) and subsequent intracellular protein kinase A (PKA) activation by the second messenger cAMP, inhibitory pathways in inflammatory T cells and macrophages are triggered (Deaglio et al., 2007).
(iii) Regarding the interactions of Treg with dendritic cells (DC) and B cells, Treg express high amounts of the inhibitory molecule cytotoxic T-Lymphocyte Antigen 4 (CTLA4), which competes for binding to CD80/CD86 with the co-stimulatory molecule CD28, thus decreasing the cross-talk between Tconv and antigen presenting cells *in vitro* and *in vivo*. In addition, Treg can also increase the expression of the enzyme indoleamine 2, 3-dioxygenase (IDO) in DCs via CTLA-4 induced signaling, resulting in starvation of Tconv and arrest of cell cycle, as IDO can catalyze degradation of the essential amino acid tryptophan, and also induces iTreg generation (Fallarino et al., 2003; Curti et al., 2007; Schmidt et al., 2012).
(iv) Granzyme, another important molecule expressed by Treg, can facilitate the killing of responder cells in a perforin-dependent manner in human and mice (Grossman et al., 2004; Gondek et al., 2008). It is also reported that the restraint of NK cell cytotoxicity by Treg is related with both granzyme B-perforin dependent manner and the limiting of IL2 availability (Cao et al., 2007; Gasteiger et al., 2013).
(v) The co-inhibitory molecule TIGIT, expressed by a distinct Treg population, specifically suppresses proinflammatory T helper 1 (Th1) and Th17 cell, but not Th2 cell responses (Joller et al., 2014).
(vi) Treg can also induce target tissue cells to express molecules like heme oxygenase 1 (HO-1), an enzyme that degrades heme. Heme is released within post-trauma hematoma, and acts as a pro-inflammatory mediator through activation of toll-like receptor 4. Thus heme degradation can reduce the inflammation dramatically (Blancou et al., 2011; Simon et al., 2011). Moreover, heme degradation further results in the formation of carbon monoxide, iron, and biliverdin, and these products also have cytoprotective and anti-inflammatory properties (Soares and Bach, 2007), which can protect endothelial cells to support angiogenesis as one of the first steps of successful tissue repair (Street et al., 2000; Simon et al., 2011).

Thus, the interaction between Treg and immune cells can create an anti-inflammatory and anti-apoptotic immune environment to promote successful tissue repair.

## Bone Tissue Regeneration

As in other tissues, successful bone tissue repair depends on a multitude of tightly regulated immune and non-immune cells and signaling molecules. However, bone repair and regeneration is becoming increasingly popular as a topic of tissue regeneration studies, as bone is able to heal without scar formation. Better understanding of the interactions between immune and bone forming non-immune cells will continue to gain importance in an aging human population. Both the skeletal and the immune systems undergo changes with aging, affecting the specific cellular potential and the interaction of both systems (Xing et al., 2010). A deeper understanding of the positive aspects of Treg could be beneficial for the emerging therapeutic needs of an aging population. Thus, the new research crossfield of osteoimmunology, has been created in recent decades (Takayanagi, 2009; Okamoto and Takayanagi, 2011; Zhao, 2012; Greenblatt and Shim, 2013).

Mounting evidence has demonstrated that both adequate innate, and balanced adaptive immune responses are necessary for successful fracture repair, independently of any immune-related disease or infection (Kolar et al., 2010; Reinke et al., 2013; Sun et al., 2014), which implies a role for Tregs in bone regeneration.

The tissue healing process has been divided into several consecutive and overlapping processes, including inflammation, repair and remodeling (Kolar et al., 2010; Schmidt-Bleek et al., 2014). During the early phase of healing, when vessels are disrupted upon bone fracture, coagulation forms a hematoma, which is marked by low pH and hypoxia, a milieu not suitable for most cells. The hematoma includes the immune cells present in the blood upon clotting. These immune cells are adapted to survive under the difficult conditions and remain active (Buttgereit et al., 2000), secreting large amounts of pro-inflammatory mediators, such as heme and cytokines like interferon gamma (IFN*γ*) and tumor necrosis factor alpha (TNF*α*; Street et al., 2000; Soares and Bach, 2007; Blancou et al., 2011; Simon et al., 2011). This pro-inflammatory reaction determines the beginning of the healing process (Kolar et al., 2010, 2011). However, the timely termination of this pro-inflammatory process is a prerequisite for the initiation of later regenerative phases such as angiogenesis and onset of endochondral ossification (Serhan and Savill, 2005; Schmidt-Bleek et al., 2012; Reinke et al., 2013). Thus, the highly controlled pro-inflammatory and anti-inflammatory phases generated by the immune system are essential to create the appropriate conditions for successful bone tissue repair. For many years research on this topic focused on innate immunity only, however more recent data supports the important role of the adaptive immunity in regeneration as well.

## Bone Homeostasis

Bone homeostasis is maintained by coordination between the processes of bone formation managed by osteoblasts (OBs), and bone resorption by osteoclasts (OCs). OCs derive from the monocyte-macrophage lineage under the effect of macrophage colony-stimulating factor (M-CSF) and receptor activator factor of nuclear factor *κ*B ligand (RANKL), which can bind with RANK on OCs precursors. This interaction can induce the fusion of OC precursors to form mature OCs. On the other hand, the bone forming cells, OBs are derived from the bone marrow mesenchymal stromal cells (MSCs) following different pathways involving several key transcription factors like Core-binding factor alpha1/Runt-related transcription factor 2 (Cbfa1/Runx2). Moreover, OBs can regulate the activity of OCs through expression of RANKL and osteoprotegerin (OPG), which can oppose the RANKL/RANK interaction (Mori et al., 2013). Balance between OBs and OCs is also heavily influenced by the immune system, mainly mediated by cytokines. Many immune cells, including T cells and B cells can produce RANKL to further promote the differentiation of OCs. Other pro-inflammatory cytokines like TNF*α*, interleukin 1 (IL1), IL6 and IL17 secreted by macrophages, T cells, natural killer cells, and neutrophils can also act on stromal cells to up-regulate the expression of RANKL and potentiate OCs differentiation. TNF*α* is the key player in the bone resorption as it can also inhibit the differentiation and bone-forming activity of OBs. The effect of IFN*γ* is rather weak in this process though it can also inhibit OC differentiation by down-regulation of TNF receptor-associated factor 6 (TRAF6). Additionally, the production of anti-inflammatory cytokines like IL4, IL10, IL35 and TGFβ by T cells, monocytes and different Treg can suppress synthesis of pro-inflammatory cytokines and inhibit OC differentiation. The effects of pro-inflammatory and anti-inflammatory cytokines on the differentiation of OBs and OCs are summarized in Table 3 (Greenblatt and Shim, 2013; Mori et al., 2013; Feng et al., 2014).

**TABLE 3:** **Pro- and anti-inflammatory cytokines secreted during tissue repair**.

**Pro-inflammatory cytokines**	**Cellular sources (immune system)**	**Effects on bone cells (bone resorption)**	**Reference**
TNF*α*	Macrophages, T cells, NK cells, neutrophils, mast cells, B cells	→ Inhibit the differentiation and bone-forming activity of osteoblasts (OB) → Promote stromal cells to express RANKL for OC differentiation → Promote OC differentiation directly	Charatcharoenwitthaya et al. (2007), Reinke et al. (2013)
IL1, IL6	T cells, macrophages, monocytes	Up-regulate RANKL to promote OC differentiation	Greenblatt and Shim (2013), Mori et al. (2013)
IL17	T cells (Th17)	→ Acts on stromal cells and OB to up-regulate RANKL and OC differentiation	Lubberts et al. (2005), Tucci et al. (2013)
IFN*γ*	NK cells, T cells	→ Inhibit OC differentiation by down-regulating TRAF6 → Indirectly effect TNF and RANKL expression → Block OB differentiation by inhibiting induction of RUNX2, a master regulator of OB differentiation	Takayanagi et al. (2000), Mori et al. (2013)
Anti-inflammatory cytokines	Cellular sources (immune system)	Major effect on bone cells (bone formation)	
IL4	T cells (Th2), mast cells, B cells, stromal cells	→ Inhibit OC differentiation → Inhibition of LPS-induced pro-inflammatory cytokine synthesis → Positively influence OB migration	Zaiss et al. (2007), Greenblatt and Shim (2013), Schmidt-Bleek et al. (2015)
IL10	Monocytes, T cells, type 1 regulatory T cells	→ Inhibit monocyte/macrophage and neutrophil cytokine production → Inhibit Th1-type lymphocyte response → Block NF*κ*B pathway	Shouval et al. (2014)
IL35	Regulatory T cells, regulatory B cells	→ Suppress the proliferation of conventional T cells → Inhibit the differentiation of Th17 cells	Egwuagu and Yu (2015), Sun et al. (2015)
TGFβ	Constitutively expressed in many cell lines	→ Inhibit monocyte/macrophage MHC class II expression → Suppress proinflammatory cytokine synthesis MHC class II expression → Suppress pro-inflammatory cytokine synthesis	Schmidt-Bleek et al. (2015)

In addition, dual-specificity phosphatase 5 (DUSP-5) is a phosphatase that specifically dephosphorylates both phosphoserine and phosphotyrosine residues of MAPK to suppress its activity. The over expression of DUSP5 in splenic CD4 T cells can decrease the number of TH17 cells and increase the frequency of Treg in mouse by modulating their key transcriptional factor STAT3 and STAT5, which was related to inhibited ERK activity (Liu et al., 2013; Moon et al., 2014). These effects can further control exaggerated inflammation and facilitate the tissue repair process. DUSP-5 was also shown to down regulate pro-osteoclastogenic molecules like RANKL, RANK, NFATc1, thus playing an important role in keeping bone homeostasis (Moon et al., 2014).

## Interaction Between Treg and Osteoclasts

So far osteoimmunology research has mostly focused on the interaction between Treg and OCs that are derived from the hematopoietic system. OCs are reported to function as antigen-presenting cells to activate CD4 T cells (Li et al., 2010). Treg have been shown to suppress OC differentiation through cell-cell contact via CTLA4, though IL4 and TGF-β were also shown to be related, but not essential to the inhibitory effect on osteoclastogenesis *in vitro* (Kim et al., 2007; Zaiss et al., 2007; Axmann et al., 2008). Meanwhile, protection of local and systematic bone destruction by Treg was observed *in vivo* (Zaiss et al., 2010a,b), indicating multiple levels of cross-talk between the skeletal and immune systems. Additionally, STAT5, an important transcriptional factor for Treg, might be another interesting modulator between OCs and Treg as it can negatively regulate the bone-resorbing function of OCs by promoting Dusp1 and Dusp2 expression (Hirose et al., 2014). However, almost no data are available on OBs.

## Possible Direct Interaction Between Treg and Osteoblasts and Their Precursors

Consistent with the interactions of Treg with other tissue cells like adipocytes and muscle satellite cells, Treg may also directly interact with bone-forming cells or their progenitor cells, the MSCs. As MSCs are the progenitor cells for many tissue cells (e.g., OBs and adipocytes) and use very similar suppression mechanisms for immune responses as Treg, they might have more intensive interactions (Glenn and Whartenby, 2014). Recently, many groups have reported that administration of MSCs can either increase the number and function of FoxP3^+^ Treg in a Jagged-1 dependent manner, or convert FoxP3^–^ Tconv into FoxP3^+^ Treg (Chao et al., 2014; Obermajer et al., 2014; Takahashi et al., 2014; Cahill et al., 2015; Cortinovis et al., 2015; Wang et al., 2015). However, the effect of Treg on MSCs is under-investigated. One group showed a positive effect on healing upon administration of combined Treg and bone marrow MSCs in a calvarial defect model in mice (Liu et al., 2011b), although, the underlying molecular mechanisms have not been revealed.

**FIGURE 3:**
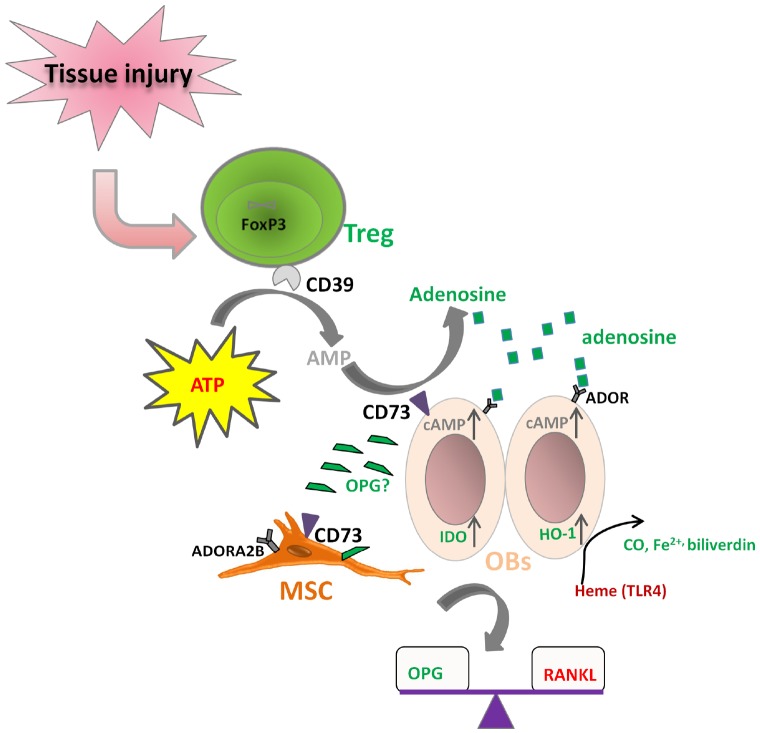
**Hypothesized direct cross-talk between Treg and osteoblasts (MSCs).** (i) CD39 expressing Treg may co-operate with CD73 expressing osteoblasts (MSCs) to hydrolyze ATP to form adenosine, which can further bind to its receptor on osteoblasts (ADOR) to trigger the inhibitory pathways; (ii) Treg may up-regulate IDO and HO-1 expression on osteoblasts; (iii) Treg play a role in the balance of RANKL/OPG, thus facilitating osteoblast differentiation.

It is known that both Treg and MSCs use the CD39–CD73-mediated adenosine-producing pathway to control inflammation. However, the dominant tTreg population mainly expresses CD39 (ENTPD1) rather than CD73 (NT5E), while bone marrow-derived MSCs mainly express CD73 rather than CD39 in human. We and others have shown that human Treg produce adenosine upon contact with CD73^+^ cells (Saldanha-Araujo et al., 2011; Schuler et al., 2014; Zhang et al., 2015). Therefore, it is possible that Treg cooperate with MSCs to convert ATP into adenosine. The resulting adenosine can signal via 4 receptor subtypes: adenosine receptor A1 (ADORA1), ADORA2A, ADORA2B, and ADORA3. Undifferentiated bone marrow-derived MSCs mainly express ADORA2B. Various receptors are important for particular differentiation outcomes. ADORA2B is the essential receptor for MSCs differentiation into OBs, enabling bone formation, while ADORA1 and ADORA2A are more related with MSC differentiation toward adipocytes (Gharibi et al., 2011; Trincavelli et al., 2014). Based on these facts, we hypothesize that Treg could act directly on OBs through coordination of the CD39–CD73-(adenosine)-ADOR pathway (Figure 3). This coordination regarding purinergic signaling may also exist between Treg and the tissue progenitor cells, which could potentiate the differentiation of MSCs and thus facilitate tissue regeneration. IDO and HO-1 induction by Treg on OBs may also be a result of direct cross-talk between Treg and OBs (Oliveira et al., 2006).

In addition, Treg may also play a role in regulating the ability of OBs to express RANKL and secrete OPG, influencing thereby the extent and degree of osteoclastogenesis. It is reported that expression of CD40L on activated CD4 T cells can induce the expression of RANKL and the suppression of secretion of OPG to facilitate osteoclastogenesis. However, Treg can inhibit CD40L expression on T cells very fast (Canavan et al., 2012; Lei et al., 2015), thus the interaction between Treg and Tconv may regulate the RANKL/OPG balance to favor OB differentiation and bone formation. Nevertheless, Treg might also induce OPG production by OBs directly in some other manner. The hypothesized direct cross-talk between Treg and OBs (MSCs) are shown in Figure 3.

## Summary or Outlook

Taken together, Treg have been shown to be resident in various tissues, with specific phenotypes and functions. They create an anti-inflammatory and anti-apoptotic environment in these tissues through the control of undesired immune activities mainly caused by other immune cells, and thus facilitate the tissue repair and regeneration process indirectly. Here, we hypothesize possible direct cross-talk between Treg and OBs (MSCs), mainly through purinergic signaling, which might also potentiate the differentiation of MSCs, and facilitate bone regeneration. This hypothesis may provide a model for further investigations on the cross-talk between the immune and skeletal system, and enable the development of better strategies to promote bone repair and regeneration.

### Conflict of Interest Statement

The authors declare that the research was conducted in the absence of any commercial or financial relationships that could be construed as a potential conflict of interest.
